# Mathematical Estimation of Axial Length Increment in the Control of Myopia Progression

**DOI:** 10.3390/jcm11206200

**Published:** 2022-10-20

**Authors:** António Queirós, Ana Amorim-de-Sousa, Paulo Fernandes, Maria Sameiro Ribeiro-Queirós, César Villa-Collar, José M. González-Méijome

**Affiliations:** 1 Clinical and Experimental Optometry Research Lab (CEORLab), School of Science, University of Minho, Gualtar, 4710-057 Braga, Portugal; 2 Physics Center of Minho and Porto Universities, (CF-UM-UP), Gualtar, 4710-057 Braga, Portugal; 3 Escola Básica Prof. Gonçalo Sampaio, 4830-523 Póvoa de Lanhoso, Portugal; 4 Departamento de Farmacia, Biotecnología, Óptica y Optometría, Universidad Europea de Madrid, 28670 Madrid, Spain

**Keywords:** axial length, keratometry, refraction, control of myopia progression

## Abstract

This study aims to evaluate the existing mathematical approach for the theoretical estimation of axial length (AL) in a cross-sectional study, developing a new mathematical model and testing it in a longitudinal sample. Many professionals do not have a device to measure the AL due to clinic space and cost of equipment. However, this parameter plays an important role in the assessment of myopia progression to monitor treatment effects with myopia control strategies. First, a cross-sectional study based on the mathematical equation proposed by Morgan was performed. The AL was estimated based on the mean values of keratometry and spherical equivalent in 1783 subjects (52% female), aged 14.6 ± 4.6 years (6 to 25 years), of whom 738 were myopic, 770 emmetropic and 275 hyperopic. On average, the AL estimated with the Morgan formula was 0.25 ± 0.48 mm larger than the real AL value (95% limits of agreement: +0.70 to −1.20 mm). The study by gender, ametropia, type of astigmatism and age showed statistically significant differences between the real AL and predicted AL_Morgan (r > 0.750, spearman). Based on the previous sample, a multiple linear regression was applied, and a new mathematical model was proposed. The model was tested on a longitudinal sample of 152 subjects whose mean age was 13.3 ± 3.1 years (9 to 24 years) and of whom 96 were female (64%). The sample consisted of 46 myopes, 82 emmetropes and 24 hyperopes. The longitudinal study of the differences in axial length at one year between the models showed no statistically significant differences and that the mathematical equations are valid for estimating differences in axial increment for ages between 9 and 24 years, despite errors in the predicted value for axial length.

## 1. Introduction

The pandemic increase in myopia in the last two decades and the predicted increase in the next two decades [1] have created an interest in the scientific community to produce guidelines for myopia control [2] and models of myopia progression with different treatments [3,4]. Several optical devices and pharmaceutical approaches with variable efficacy have been tested to control myopia progression [2]. Since the rate of progression of eye growth is related to age [5] and ametropia [6], clinical treatment to control myopia progression requires knowledge of the rate of the axial eye length increment [7]. Thus, rates of myopia progression control have been presented based on a slower eye growth rate from 30 to 80% [7].

Objectively, the eye length value can be measured using ultrasound techniques (invasive measurement with local anesthesia) or optical coherence biometers (noninvasive measurements). However, these types of equipment are professionally limited to clinics and hospitals due to their economic value and technical specification for use. Despite the efforts made by the industry and scientists, applying these techniques is not universal by professionals worldwide [8]. With the implementation of well-defined protocols and rules, the lack of instrumentation is a limitation to monitoring myopia progression. Moreover, the axial length measurement is one of the most relevant factors that should be considered more carefully when myopia increases [9].

Mathematical models have been proposed to estimate the eye’s axial length based on variables easily obtained, such as age, refraction and keratometry [6,10,11]. With the present work, from the proposed model, the authors intended to present an easy, inexpensive and accessible way for all eye care professionals to obtain axial length when it is not possible to be measured directly and thus enable a better management in the control of myopia progression.

Thus, this study aimed to apply a mathematical model to estimate axial length in a population of children and young people in Portugal and propose a new mathematical model, compare it to the previous model and understand it in longitudinal terms by estimating changes in axial length.

## 2. Material and Methods

### 2.1. Subjects

A total of 1783 children and young subjects were included: 870 males and 913 females (mean age, 14.6 ± 4.6 years; range, 6–25 years). All teenagers were students at School 2/3 de Caldas das Taipas, Guimarães, Portugal, and young students at the University of Minho. All measurements were obtained before 2019. The protocol and study procedures were reviewed and approved by the Ethics Subcommittee for Health and Life Sciences of the University of Minho. The study followed the principles of the Declaration of Helsinki. All participants or their parents (in the case of children) provided written informed consent after they received an explanation of the nature, procedures and consequences of the study.

### 2.2. Measurements

The central refractive error was assessed with an open-field instrument, the Grand Seiko Auto Ref/Keratometer WAM- 5500 (Grand Seiko Co., Ltd., Hiroshima, Japan), without cycloplegia and with a fixation point at 6 m distance [12]. The average value of five consecutive measurements of the refractive error (sphere, cylinder and axis) in the right eye was included. In order to facilitate the analysis of refractive error, the vectorial components mentioned by Thibos et al. (M, J0 and J45) were calculated and used for analysis [13].

Values of eye’s axial length (AL) and keratometry (Kx, as mean of Kflat and Ksteep) were obtained using the biometer IOL Master 500 (Carl Zeiss, Jena, Germany). The subjects were instructed to fixate the fixation point within the IOL Master. The average of three measurements was registered.

The values obtained from keratometry and central refractive error from 1783 eyes were used to predict the axial length in a young Portuguese population, using the mathematical model presented by Morgan et al. [10].

Morgan formulae:

AL_Morgan = 1/[(0.22273/Kx) + 0.00070 × S + 0.01368]

(S = spherical equivalent refractive error at the corneal plane (D)).

For the validation of the model, the previous measurements were repeated after one year (370 ± 15 days) in 152 subjects. None of these participants were contact lens users or had received any treatment for myopia control in this period.

### 2.3. Statistical Analysis

The statistical package SPSS v.21 (SPSS Inc., Chicago, IL, USA) was used to conduct the statistical analysis. The Kolmogorov–Smirnov test was applied to evaluate the normality of data distribution. The Mann–Whitney U-test or Kruskal–Wallis test was used to analyze the differences in variables M, keratometry, AL_Real and AL_Predicted depending on gender (male or female), sphere value (sphere of refraction ≤ −1.00 or >−1.00 D), ametropia (myopia for M ≤ −0.50 D, emmetropia for −0.50 < M < +0.50 D and hyperopia for M ≥ +0.50 D), type of astigmatism (astigmatism with-the-rule for cylinder 0°/180° ± 20°, astigmatism against-the-rule for cylinder 90° ± 20° and oblique for the rest) and age (age groups: 6–9 years, 10–12 years, 13–17 years and ≥ 18 years).

The AL values from the biometer and those calculated were correlated and analyzed through Spearman’s correlation, and the Bland–Altman analysis was performed to compare these values. Multiple linear regression was used to study the variables and develop a new mathematical model that was later tested using the ROC curves (receiver operating characteristic).

A *p*-value < 0.05 was accepted as statistically significant.

## 3. Results

**Figure 1:**
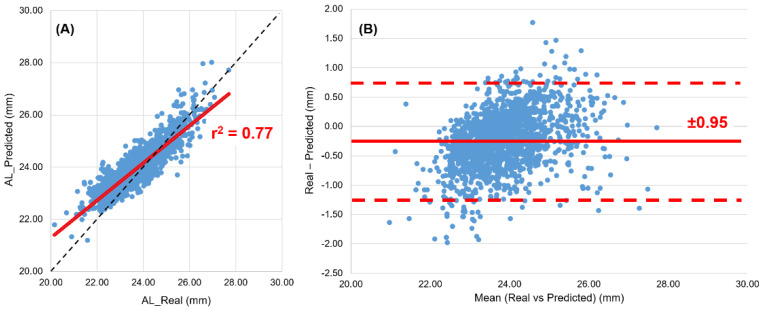
Linear regression (**A**) and Bland–Altman (**B**) analysis between AL_Real (obtained with IOL master) and AL_Predicted (calculated using the Morgan model).

A total of 1783 subjects (aged 6 to 25 years, of which 913 were female) were analyzed to test Morgan’s formula. The mean value of the spherical equivalent (M) refraction was −0.78 ± 1.67 D, with sphere ranging from −8.25 D to +8.50 D and maximum −4.00 D astigmatism. The average keratometry was K = 7.77 ± 0.28 mm. A total of 41.4% of the subjects were myopes (M = −2.34 ± 1.45D), 43.2% emmetropes (M = +0.08 ± 0.26D) and 15.4% hyperopes (M = +0.97 ± 0.65D) according to the criteria defined previously. By comparing the AL measures with IOL (AL_Real) versus the AL predicted with the Morgan formulae (AL_Predicted), an interclass correlation coefficient (ICC) of 0.830 [0.707 a 0.891, *p* < 0.001] was found (Figure 1A). The Bland–Altman plot shows that, on average, the AL_Predicted values are 0.25 mm ± 0.48 mm larger than the AL_Real values, with 95% limits of agreement between +0.70 mm and −1.20 mm (Figure 1B). However, by the analysis of Figure 1B, Morgan’s formula shows a proportional bias. The graphical analysis of the best fit line clearly shows larger predicted values (Morgan’s formula) than the real ones in shorter eyes and the opposite in longer ones. The same analysis shows that the real value is closer to the predicted value (differences close to zero) when the eyes have an axial length around the 25 mm average axial length.

Table 1 shows the values of the mean objective spherical equivalent, keratometry and AL value obtained with the IOLMaster. Additionally, the value of AL_Predicted calculated using Morgan’s formula and its difference with the AL_Real is also presented. The differences between the measured value and the calculated value of AL as a function of gender, sphere value of refractive error, ametropia, type of astigmatism and age of the subjects are also presented in this table. Overall, the average measurements for the AL_Predicted were consistently higher than AL_Real (*p* < 0.001, Wilcoxon test). This difference ranged from a minimum of 0.61% (−0.15 ± 0.46 mm) in subjects with a sphere ≤ 1.00 D to a maximum of 2.11% in children aged 6–9 years (−0.50 ± 0.53 mm). The lowest values in Morgan’s formula difference happened for males, sphere ≤ 1.00 D, myopic and subjects of 18–25 years. The study of the correlations found showed statistical significance for all of them being higher than 0.75 (*p* < 0.001, Spearman correlation).

New AL prediction model—the same sample was used after the Morgan et al. formula analysis to study a new mathematical model based on data obtained without cycloplegic refraction.

A multiple linear regression analysis was performed to find the factors that most influence axial length (dependent variable). The model found statistically significant variables of age, sphere equivalent and keratometry, and these were included as independent variables.

The established linear regression suggests that age, M (spherical equivalent, D) and Kx (mean of keratometry, mm) could statistically significantly predict AL—F (3,1766) = 2323.65, *p* < 0.001) and accounted for 79.8% of the explained variability in AL with the following predictive relationship:

AL_Queiros = 0.019 × Age + 2.271 × Kx − 0.444 × M + 5.414

The model was tested on a longitudinal sample of 152 subjects observed over a 1-year interval (370 ± 15 days), with the criteria defined in Table 2. The mean age was 13.3 ± 3.1 years (9 to 24 years), of which 96 were female (64%). The sample consisted of 46 myopes, 82 emmetropes and 24 hyperopes. Apart from the keratometry values, significant increment values were found at one year for refraction (more myopic by 0.15 ± 0.42 D) and axial length (+0.067 ± 0.125 mm growth). Although there are statistically significant differences between the baseline and the values obtained after one year, the increments are not statistically significant (*p* = 0.241 Kruskal–Wallis Test), and there is a high correlation between the values measured at baseline and after one year (r > 0.981, *p* < 0.001, Spearman’s r).

**Figure 2:**
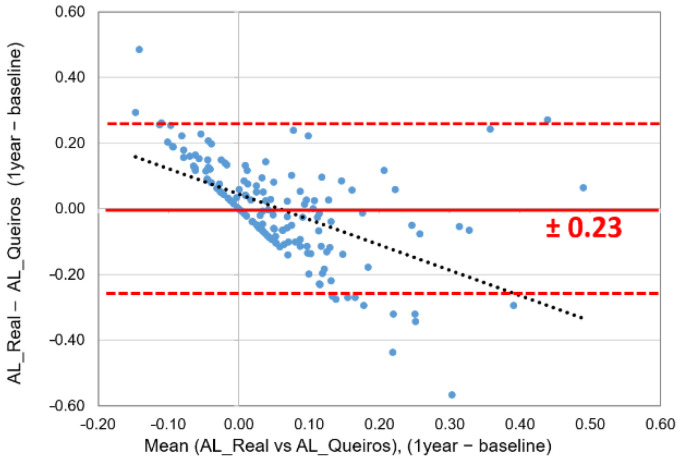
Bland–Altman plot for the 1-year differences between the real measured value of AL and this study.

The analysis of the differences between the initial measurement and over one year for the value calculated by the new formulation and the actual value shows that, on average, the value is zero with 95% limits of agreement between ±0.23 mm (Figure 2). However, a more promising data analysis indicates that in 53% of the cases, these differences are smaller than 0.10 mm, 74% smaller than 0.15 mm, and in 82%, these differences are 0.20 mm.

**Figure 3:**
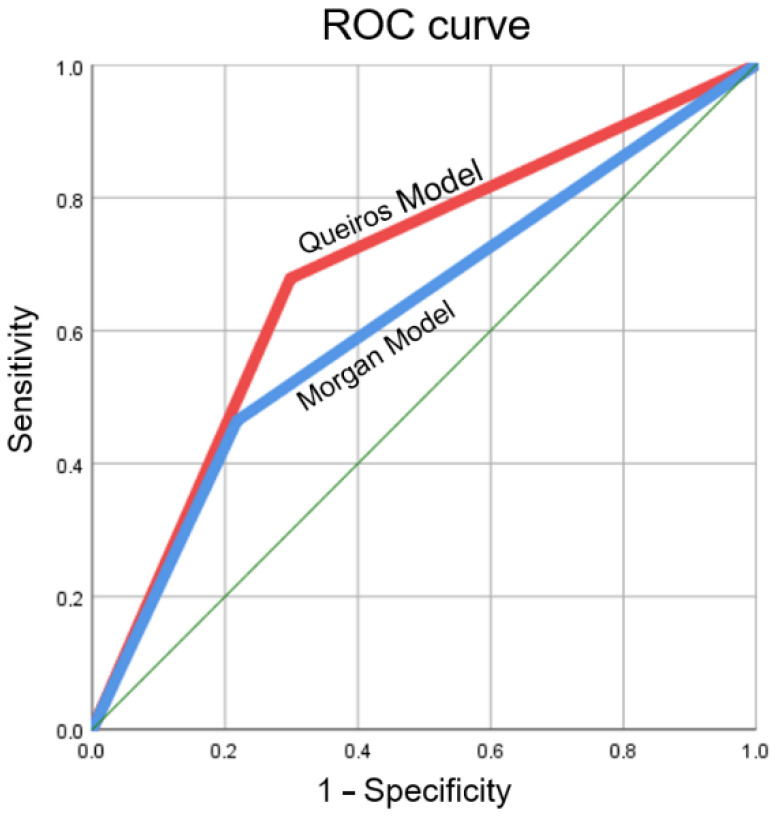
Receiver operating characteristic curves (ROC) for increment differences of 0.10 mm in axial length detected by Morgan’s equation (Morgan Model) and the present study (Queiros’s Model).

In order to test the new model, the receiver operating characteristic (ROC) curves were constructed to analyze sensitivity and specificity in detecting 0.10 mm increments in axial length at one year. In order to compare the models, Morgan’s equation was applied to the same graphical construction. The area under the receiver operating characteristic curve (AUROC) was calculated (Table 3, Figure 3).

## 4. Discussion

Estimating the axial length of the eye using mathematical equations may be useful for vision care professionals to control the progression of myopia when it is not possible to obtain it using appropriate equipment. The analysis of Morgan’s formula showed that the estimated value for AL (AL_Predicted) presents, on average, larger values of 0.25 ± 0.48 mm than the real value (AL_Real). This difference is more accurate in males, myopic patients and children older than 12 years. Moreover, the correlations between the measured and calculated AL values were strong (r > 0.750). Despite these errors in the longitudinal analysis, we found no statistically significant differences between the calculated and measured axial length value at 1 year (delta AL). Nevertheless, although the mean of the differences is zero (yy-axis), Figure 2 shows a clear tendency for the formula to estimate lower axial eye increment over one year than the real increment value (AL_Queiros < AL_Real) when the mean of the longitudinal changes (xx-axis) is negative and the opposite when the mean of the longitudinal changes is positive.

Axial length measurement has become a key element in both the evaluation of axial myopia and in monitoring the progression and evaluating the effectiveness of existing treatments to control myopia progression. A recent paper by Morgan et al. described the relationship between cycloplegic refraction and keratometry. Their formula for axial length calculation was based on data from 144 children aged 8–12 years followed for 36 months in a myopia progression control study with contact lenses (dual focus, daily disposable soft contact lenses). According to the authors, the formula was validated on two different samples and shows, on average, axial length values zero [14] and 0.13 mm [15] greater than the measured, with a 95% confidence interval and agreement of error values from ±3.0% (±0.75 mm) to ±3.7% (±0.75 mm), respectively. Galvis et al. found higher values in a study of Colombian children aged 8–17 years, where the AL_calculated was, on average, 0.52 mm longer than the AL_Real with a range of agreement of ±4.2% [16]. In the current study, we found values of 95 % confidence limits of ±3.9% (±0.95 mm), with the AL_calculated greater than the AL_measured by 0.25 ± 0.48 mm.

In contrast, Morgan et al. emphasize the importance of the formula to reasonably predict the value of axial length, although they point out that the margin of error may compromise the longitudinal follow-up of AL changes in myopes. The longitudinal analysis of the present study showed no statistically significant differences in both the AL obtained using Morgan’s formula and that obtained through the formula presented in this study compared to the actual axial length increments (Table 2). Although the predicted value deviates from the actual value of AL, it was also possible to verify that both formulas detected differences superior to 0.10 mm in axial increment at one year with reasonable sensitivity and specificity. For myopia progression management, the amount of axial growth over a while is more important than the change in the magnitude of the refractive error itself. The stretching of the tissues due to axial elongation may have potential future implications for visual health and quality of life.

The fact that the formulae have not been tested in populations with different myopia control treatments is one of the limitations of this study. Thus, and given that these treatments slow the eye growth by an average of 50%, it will be important to verify the formulae’s effectiveness. This becomes even more relevant due to two aspects shown in Table 1 regarding the differences AL_Real—AL_ Predicted. First, in these studies, the populations are composed only of myopes, where the differences are smaller (−0.16 ± 0.78 mm) compared to emmetropes and hyperopes. Secondly, and on the other hand, this formula may fail when applied in these myopia control studies since, at younger ages (6–9 years), where these treatments should preferably start, the difference is higher (−0.50 ± 0.53 mm) due to the developing emmetropization process. In addition, it is further difficult to estimate the eye’s axial length at such ages due to the impact of changes occurring in the lens compared to older eyes [17,18,19,20,21,22]. However, despite these limitations, the validation was performed on subjects aged 9 to 24 years, with 65% of the sample consisting of subjects between 9 and 13 years old, and should be validated on larger samples considering both the age factor and the error factor in the repeatability of the instrument IOL Master (±0.010 mm) [18,23].

Another limitation of this study is related, or not, to the acquisition of the refraction value of the subjects. While the Morgan et al. formulation considers cycloplegic refraction (important in controlling accommodation, especially in children), in the present study, using an open-field autorefractometer with fixation at 6 m minimized this effect [12]. As such, the new model has an advantage in obtaining the noncycloplegic refraction of subjects since its use is forbidden by most optometrists worldwide. However, the criteria used to obtain the refraction should always be considered in the AL estimation formula.

Although this study presents an alternative for calculating the axial length, it is not of interest to replace the performance of objective measurements of axial length. This type of mathematical model may be helpful for eye care practitioners without access to specialized equipment for measuring the axial length and to whom the use of cycloplegic drugs is not authorized, even though the proposed model does not estimate an exact value of axial length. Compared to earlier formulations with the same purpose, the model proposed in this study includes the subjects’ ages as a differential factor to estimate the longitudinal changes in axial length by using the estimated eye length value from some visits, even though this is not the real AL value. When appropriate equipment for the objective measurement of axial length is not available, the possibility of accessing an estimated value from data usually recorded during a refractive examination by eye care professionals, such as those used in the model proposed in this study (age, refraction and keratometry, whether via videokeratoscopy, autokeratometer or manual keratometer), may be of great help to the decision-making process in myopia management in children, especially when retrospective data from patients are available. In this sense, the longitudinal analysis of the model proposed may help those clinicians to estimate the amount of eye growth over a period of time and to be able to intervene, together with parents, to control myopia progression beforehand. Thus, by including the child’s age, the refraction measurement and the keratometry value, the clinician can estimate axial eye length increment values retrospectively from medical recordings and thus obtain additional information in clinic appointments in a less empiric way. Moreover, it may allow the clinician to determine the appropriate moment of intervention to start the treatment to control myopia progression using one of the many modalities currently available for this purpose.

## 5. Conclusions

This work showed that the formula of age, spherical equivalent and mean keratometry allows eye care professionals to estimate the difference in axial length increment longitudinally for ages between 9 and 24 years and may be a good alternative for clinicians who cannot perform this measurement in the control of myopia progression. However, this estimation should be minimally informative for eye professionals and does not replace the axial length measurement for surgical purposes.

## Figures and Tables

**Table 1:** Objective spherical equivalent refraction (M), mean keratometry (Kx), ocular axial length (AL_Real) and predicted ocular axial length (AL_Predicted) presented in mean ± SD, according to gender, sphere of refractive error, ametropia, type of astigmatism and age group.

	n	M (D)	K (mm)	AL_Real (mm)	AL_Predicted (mm)	AL_Real—AL_Predicted (mm)	Correlation Real vs. Predicted
Male	870	−0.70 ± 1.56	7.80 ± 0.28	23.74 ± 0.99	23.95 ± 0.81	−0.20 ± 0.46 (¥)	r = 0.873 (§)
Female	913	−0.86 ± 1.76	7.75 ± 0.28	23.62 ± 1.01	23.91 ± 0.82	−0.29 ± 0.50 (¥)	r = 0.863 (§)
Sphere ≤ −1.00	562	−2.78 ± 1.38	7.68 ± 0.26	24.41 ± 0.90	24.56 ± 0.84	−0.15 ± 0.46 (¥)	r = 0.839 (§)
Sphere > −1.00	1221	0.14 ± 0.70	7.81 ± 0.28	23.34 ± 0.86	23.63 ± 0.62	−0.29 ± 0.49 (¥)	r = 0.811 (§)
Myopia	738	−2.34 ± 1.45	7.70 ± 0.28	24.25 ± 0.94	24.42 ± 0.86	−0.16 ± 0.78 (¥)	r = 0.854 (§)
Emmetrope	770	+0.08 ± 0.26	7.81 ± 0.27	23.37 ± 0.80	23.66 ± 0.57	−0.29 ± 0.47 (¥)	r = 0.792 (§)
Hyperope	275	+0.98 ± 0.65	7.83 ± 0.27	22.97 ± 0.83	23.34 ± 0.57	−0.37 ± 0.52 (*)	r = 0.750 (§)
With-the-Rule	856	−0.73 ± 1.76	7.77 ± 0.28	23.64 ± 1.03	23.92 ± 0.85	−0.28 ± 0.47 (¥)	r = 0.883 (§)
Oblique	266	−0.70 ± 1.60	7.79 ± 0.29	23.65 ± 0.92	23.94 ± 0.79	−0.28 ± 0.46 (¥)	r = 0.845 (§)
Against-the-Rule	375	−0.81 ± 1.65	7.76 ± 0.31	23.67 ± 1.01	23.91 ± 0.82	−0.24 ± 0.48 (*)	r = 0.864 (§)
6–9 years	262	−0.44 ± 1.56	7.75 ± 0.25	23.24 ± 0.94	23.74 ± 0.73	−0.50 ± 0.53 (¥)	r = 0.818 (§)
10–12 years	437	−1.50 ± 1.67	7.70 ± 0.28	23.87 ± 1.00	24.07 ± 0.86	−0.21 ± 0.47 (¥)	r = 0.874 (§)
13–17 years	433	−0.64 ± 1.49	7.79 ± 0.27	23.69 ± 0.96	23.92 ± 0.76	−0.23 ± 0.45 (¥)	r = 0.860 (§)
≥18 years	651	−0.53 ± 1.68	7.80 ± 0.30	23.72 ± 1.00	23.91 ± 0.84	−0.19 ± 0.46 (¥)	r = 0.882 (§)

* Kruskal–Wallis test, ¥ Wilcoxon, § r of Spearman.

**Table 2:** Changes of the AL_Queiros equation compared to the AL_Morgan equation and the real value of AL, when compared longitudinally for a sample of 9 to 24 years old. Values are presented as mean ± SD.

	M (D)	K (mm)	AL_Real(mm)	AL_Morgan(mm)	AL_Queiros (mm)	Pairwise
Baseline	−0.59 ± 1.66	7.73 ± 0.26	23.44 ± 0.91	23.78 ± 0.82	23.50 ± 0.88	1–0; 1–2
After 1 year	−0.75 ± 1.72	7.72 ± 0.26	23.50 ± 0.93	23.83 ± 0.86	23.56 ± 0.91	1–0; 1–2
difference	−0.15 ± 0.42	−0.01 ± 0.06	+0.067 ± 0.125	+0.043 ± 0.163	+0.066 ± 0.178	*p* = 0.241 *
*p*	<0.001 ¥	0.149 ¥	<0.001 ¥	0.005 ¥	<0.001 ¥	
Correlation	r = 0.970, <0.001 §	r = 0.970, <0.001 §	r = 0.991, <0.001 §	r = 0.982, <0.001 §	r = 0.981, <0.001 §	

* Kruskal–Wallis Test, ¥ Wilcoxon, § r of Spearman. Pairwise test (0—Real, 1—Morgan and 2—Queiros).

**Table 3:** Diagnostic efficacy of axial length calculation. The mean area under the curve and its range is presented.

	Area under the Curve	*p*	Sensitivity	1—Specificity
Morgan et al. [10]	0.623 [0.501 to 0.744]	0.042	0.464	0.218
This study	0.690 [0.580 to 0.801]	0.002	0.679	0.298
